# Curriculum implementation challenges: Development and validation of an integrated curriculum implementation challenges tool

**DOI:** 10.12669/pjms.40.1.7258

**Published:** 2024

**Authors:** Kinza Aslam, Rehan Ahmed Khan, Mohammad Annas Aslam, Fatima Zia Zaidi

**Affiliations:** 1 Kinza Aslam, BDS, MMEd, Department of Medical Education, The University of Lahore, Lahore, Pakistan; 2 Rehan Ahmed Khan, MBBS, FCPS, FRCS, PhD HPE Department of Surgery & Department of Medical Education, Riphah International University, Rawalpindi, Pakistan; 3 Mohammad Annas Aslam, BDS, MSc Department of Oral Medicine, Rashid Latif Dental College, Lahore, Pakistan; 4 Fatima Zia Zaidi, MBBS, MPhil, MMEd, Department of Medical Education, The University of Lahore, Lahore, Pakistan

**Keywords:** Curriculum, Faculty, Dentistry, Surveys and Questionnaires, Challenges, Education, Medical

## Abstract

**Objective:**

To develop an instrument to identify the challenges faced by faculty while implementing an integrated curriculum in an undergraduate dentistry program.

**Methods:**

The study was conducted between September 2020 and October 2021 at the University College of Medicine and Dentistry (UCMD), University of Lahore (UOL). A preliminary questionnaire, developed through literature review and faculty interviews was sent to 10 medical education experts for content validation via the Delphi technique. Content Validity Index (CVI) was calculated for individual items (I-CVI) as well as for the composite scale (S-CVI). A panel agreement of more than 75% was considered as the criterion for the inclusion of items in the questionnaire. Cognitive pretesting of five faculty members was conducted and pilot testing was subsequently done with 27 faculty members. The reliability of the tool was determined by Cronbach’s alpha.

**Results:**

After the Delphi process, the final Integrated Curriculum Implementation Challenges (ICIC) questionnaire had 42 items. S-CVI was 0.87 and the cut-off value for I-CVI was taken as 0.78 as the criterion for item deletion. Cognitive interviews and pretesting revealed good item interpretation. Cronbach’s alpha for this tool was 0.87.

**Conclusion:**

ICIC is a useful instrument with good reliability and content validity. It can be used to identify the presence and extent of challenges faced by the faculty while implementing an integrated curriculum.

## INTRODUCTION

Education must change with time and adapt to present circumstances. To produce competent healthcare professionals (HCPs) and in response to guidelines issued by various accrediting bodies, the medical curriculum has also evolved.1 The challenges of the evolved curriculum are met by integration, by valuing students’ prior knowledge and by using it as a starting point to build upon.2,3

In 2010, to keep up with the changing needs of society and international accrediting bodies, Pakistan Medical and Dental Council (PM&DC) declared that all medical and dental colleges implement an integrated curriculum (IC). Various studies also suggested the need for modern teaching-learning methodologies as part of IC to produce competent HCPs in Pakistan.4 Following the guidelines of PM&DC as well as the recommendations found in the literature, UCMD, UOL implemented IC.

However, initiating and implementing this change was not easy as the dental curriculum has historically been dominated by individual departments that control what should be taught, how it should be taught, and who it will be taught by.5 In addition to this, implementation of a new curriculum necessitated a change in the method of instruction and assessment which required a continuous, dedicated effort by faculty.6 While several instruments were available to evaluate difficulties associated with the curriculum, no validated instrument was found to identify the challenges of implementing IC in dentistry from the faculty’s perspective. This study aimed to develop and validate such an instrument.

## METHODS

After obtaining approval from the Ethical Review Board (ERC 9/20/03-B), the study was conducted between September 2020 and October 2021 at the School of dentistry, UCMD, UOL. A mixed method, sequential exploratory design was used for the development and validation of this questionnaire. Areas of difficulty pertinent to implementation phase of IC were identified using faculty interviews, as well as an extensive review of literature that went beyond literature pertinent to medical and dental colleges only. A preliminary questionnaire based on 48 questions was prepared for content validation via the Delphi technique.

Ten medical educationists qualified in medical education/health professions education, who were working in medical and dental institutes with IC, were approached for this stage of questionnaire development. They were sent a web-based questionnaire along with a content validity form to grade the relevance of items on a five-point Likert scale. A panel agreement of more than 75% was considered as the criteria for inclusion and exclusion of items. Based on the results of the first round of Delphi, the questionnaire was modified and re-sent to the same experts for round two of validation.

A panel agreement on all items was achieved in the second round. The content Validity Index for individual items (I-CVI) as well as for the scale (S-CVI) was calculated using the ratings of item relevance by experts in the second round. Through convenience sampling, five faculty members were selected for cognitive interviews. This was done to check the questionnaire for faculty understanding. Individual interviews were conducted, and concurrent verbal probing technique was employed during the process.^4^ The criteria of cognitive validity included: comprehension of the concept, coherent elaboration, and congruent answer. The final questionnaire was given to 27 faculty members to establish its reliability.

## RESULTS

After an extensive literature review and faculty interviews, six areas of difficulty were identified: Working environment, distribution of workload, communication, faculty development and retention, evaluation and leadership. Based on these areas, a preliminary questionnaire of 48 items was developed. These questions were sent to experts for content validation via the Delphi technique. Table-I summarizes the results of the Delphi rounds. In the first round, six out of ten (n=60%) panelists returned the preliminary questionnaire.

**Table-I:** Summarized results of the Delphi rounds

Round	Total Items	Items Deleted	Unsure but retained for following stage
1	48	2	5
2	46	4	0

Based on the results, two items were deleted and five were modified and sent back to the same panelists for validation. After round two, four items were deleted and one was retained making a total of 42 questions, under six domains, (Table-II). The content validity index of individual items (I-CVI) as well as of the composite scale (S-CVI) was calculated. S-CVI was calculated with the S-CVI/Avg method, (Table-III). This was followed by the conduction of cognitive interviews to yield an in-depth analysis of response processes and to inform item revision.

**Table-II:** ICIC tool showing subdomains with allocated itemsitems.

***Working Environment***					
1. The environment of the workplace was conducive for the implementation of integrated curriculumcurriculum.					
2. The learning environment was enthusiastic hence formed a supportive learning communitycommunity.					
3. The process of conflict resolution was skillfulskillful.					
4. The process of conflict resolution was cordialcordial.					
5. The faculty showed no reluctance towards the implementation of integrated curriculum.					
6. The Inter DepartmentalInter-Departmental (MBBS and BDS) collaboration was smooth despite the campuses being geographically distant.					
***Distribution of workload***					
7. Faculty was motivated to implement the integrated curriculum due to clear definition of their roles and responsibilities.					
8. Faulty was motivated to implement the integrated curriculum due to even distribution of workload.					
9. Faculty was able to fulfill their professional duties due to even distribution of workload.					
***Communication***					
10. The communication amongst team members was timely.					
11. The communication amongst team members was meaningful.					
12. The communication amongst team members was shared in a variety of formats (written, verbal etc.)					
13. Face to face communication amongst team members was a regular feature during the implementation phase.					
14. Sharing reflections was a regular feature during the implementation phase.					
15. A student affairs department was in place to ensure good communication between the faculty and students.					
16. The student affairs department ensured timely communication regarding matters pertinent to integrated curriculum, to the students.					
17. The student affairs department ensured timely communication regarding matter matters pertinent to integrated curriculum, to the parents.					
18. The student affairs department conducted student orientation programs to facilitate the acceptance of integrated curriculum.					
***Faculty Development and Retention***					
19. The organization/institution was attentive to the needs of the faculty (e.g. adequate staffing).					
20. Training support was provided to the faculty by means of a sound faculty development program.					
21. Faculty was were trained in a way that helped them overcome their apprehensions regarding the integrated curriculum.					
22. Faculty development sessions gave the teachers an opportunity to model the new teaching/learning strategies being introduced.					
23. Sufficient staff was trained to identify the content for individual courses in the integrated curriculum.					
24. Sufficient staff was trained to identify the learning outcomes for the individual courses in the integrated curriculum.					
25. Faculty was were trained to overcome barriers such as time constraints to ensure effective implementation of integrated curriculum.					
26. Sufficient facilitators were trained to implement the proposed teaching/learning strategies (e.g. PBL, Small Group Discussions etc.)					
27. Faculty was were trained to identify a customisedustomized set of assessment principles for integrated assessment.					
28. Faculty was were trained to implement an integrated assessment system.					
29.Faculty was were trained to develop an assessment system that matched the new teaching methodologies.					
30. Faculty was were trained to help the students overcome any apprehensions they had about the integrated curriculum.					
31. Faculty was were trained to correlate the theoretical underpinnings with the practical implementation of integrated curriculum.					
32. A reward structure was in place to compensate for the increased work loadworkload on faculty.					
***Evaluation***					
33. SWOT (Strength, Weakness, Opportunity Threat) Analysis, to evaluate the status of implementation was conducted regularly.					
34. Gap analysis was conducted during implementation phase to identify missing links between course content.					
***Leadership***					
35. Leadership exhibited a sound knowledge of the theoretical underpinnings of the integrated curriculum.					
36. Multi professionalMulti-professional leadership was in place during the implementation phase.					
37. Leadership communicated the organization’s mission/vision clearly.					
38. A transparent reporting system was present in the form of a clearly established hierarchy of leadership/administration.					
39. Leadership was able to convince the faculty of the importance of the shift in the curriculum (from traditional to integrated).					
40. Leadership implemented the integrated curriculum after consultation with faculty.					
41. Leadership did not implement the integrated curriculum merely due to external pressure from the accrediting authorities.					
42. Leadership provided adequate support to faculty in terms of good infrastructure (library, skill labs, PBL rooms etc.)					

All statements will be marked using the following Likert scale:					
	***Strongly Disagree***	***Disagree***	***Neutral***	***Agree***	***Strongly Agree***
	1	2	3	4	5

**Table-III:** I-CVIs and S-CVI/Avg for Delphi Round-2.

Questions	Expert 1	Expert 2	Expert 3	Expert 4	Expert 5	Expert 6	No. of experts with desired rating	Total no. of experts	CVI
1	R	R	R	R	R	R	6	6	1
2	R	R	R	R	R	R	6	6	1
3	R	R	R	NR	R	R	5	6	0.83
4	R	R	R	NR	R	R	5	6	0.83
5	R	R	R	R	R	R	6	6	1
6	R	R	R	NR	R	R	5	6	0.83
7	R	R	R	R	R	R	5	6	0.83
8	R	R	R	R	R	R	5	6	0.83
9	R	R	R	R	R	R	6	6	1
10	R	R	R	R	R	R	6	6	1
11	R	R	R	R	R	R	6	6	1
12	R	R	R	R	R	R	6	6	1
13	R	R	R	R	R	R	6	6	1
14	R	R	R	R	R	R	6	6	1
15	R	R	R	NR	R	R	5	6	0.83
16	R	R	NR	R	R	R	5	6	0.83
17	R	R	R	R	R	R	6	6	1
18	R	R	R	R	NR	R	5	6	0.83
19	R	R	R	NR	R	R	5	6	0.83
20	R	NR	R	R	R	R	5	6	0.83
21	R	R	R	R	R	R	6	6	1
22	R	R	R	R	R	R	6	6	1
23	R	R	R	R	R	R	6	6	1
24	R	R	R	R	R	R	6	6	1
25	R	R	R	R	R	R	6	6	1
26	R	R	R	R	R	R	6	6	1
27	R	R	R	R	R	R	6	6	1
28	R	R	R	R	R	R	6	6	1
29	R	R	R	R	R	R	6	6	1
30	R	R	R	R	R	R	6	6	1
31	R	R	R	R	R	NR	5	6	0.83
32	R	R	NR	R	R	R	5	6	0.83
33	R	R	R	NR	R	R	5	6	0.83
34	R	R	R	NR	R	R	5	6	0.83
35	R	R	R	R	R	R	6	6	1
36	NR	R	R	R	R	R	5	6	0.83
37	R	NR	R	R	R	R	5	6	0.83
38	R	R	R	NR	R	R	5	6	0.83
39	R	R	R	R	R	R	6	6	1
40	R	R	NR	R	R	R	5	6	0.83
41	NR	R	R	R	R	R	5	6	0.83
42	R	R	R	NR	R	R	5	6	0.83

S-CVI /Avg : 0.87, R: Relevant, NR: Not Relevant.

### Cognitive pre-testing

No changes were made to the questionnaire as a result of cognitive pre-testing as all respondents showed good item comprehension and elaboration along with congruent answer choices.

### Pilot testing

To establish the reliability of the instrument, the final version of the questionnaire was distributed to 30 faculty members out of which 27 (n=90%) returned completed copies. The results were entered into SPSS 20 to calculate the reliability of the scale and its subdomains, (Table-IV).

**Table-IV:** Cronbach’s alpha for domains and full ICIC tool.

Domain	No. of items	Cronbach’s alpha
Working Environment	6	0.73
Distribution of workload	3	0.73
Communication	9	0.65
Faculty Development and Retention	14	0.80
Evaluation	2	0.60
Leadership	8	0.60

**Full questionnaire scale**	**42**	**0.87**

## DISCUSSION

Curriculum change is inevitable and a need for dental IC has also been identified locally, in Pakistan.7 While there are several blueprints available that can guide institutes through the development of IC, the literature does not report any validated tool that can be used to identify the challenges of implementing said curricula.8 Hence, following the guidelines laid down in AMEE guide 87, the ICIC instrument was developed through a mixed methods approach.^9^ After a literature review and faculty interviews, many challenges associated with the implementation of an IC were identified. These challenges were grouped under six themes: Working environment, distribution of workload, communication, faculty development and retention, evaluation and leadership.

Regarding the first theme, working environment, faculty believed the implementation of IC had an impact on working environment since it increased the need for departmental interaction and coordination, which was challenging because not all faculty members were on the same page about implementation. Most faculty members were apprehensive because they believed integration would ultimately lead to a loss of departmental autonomy which is consistent across the literature as well.10 Regarding the second theme, distribution of workload, 100% of the faculty members reported being demotivated due to an exponential increase, and uneven distribution of, workload in the absence of financial incentive or compensation.

Literature also reports faculty burnout due to an uneven distribution of workload in the absence of proper funding for capacity building.11,12 Communication emerged as the third theme, the lack of which was identified as a major barrier to implementation of new curriculum. Literature also reports that absence of proper communication can leave the faculty feeling ill-equipped to handle the task of curriculum implementation and can hence cause delays in implementation process.13 The fourth and fifth themes to emerge from the interviews were faculty development and retention respectively.

All faculty members agreed that ample workshops were conducted for their training. However, there was no evaluation process to monitor the effectiveness of the workshops or IC implementation. A continuous faculty development program coupled with a strong plan for program evaluation is integral not only for professional development of staff but also for their retention and stability in implementation process.14,15 The sixth and final theme to emerge was leadership. Faculty believed it was the leadership’s responsibility to ensure adequate resources were available and communication channels between departments were fluent for IC implementation.

Literature also emphasizes the vital role educational leaders must play to nurture systems that are capable of developing HCPs who are ready to tackle the challenges of tomorrow.16 After a thematic analysis of the interviews, a total of 48 statements were generated under different themes which were then sent to experts for validation for which a cut-off consensus value of >75% was determined beforehand. After an iterative process and two rounds of Delphi, 42 items remained. The content validity index of items as well as the scale was calculated using the ratings of item relevance by these experts (Table-III). For 6-10 experts, a minimum I-CVI of 0.78 is acceptable and an S-CVI of 0.8 is desirable if calculated using the average method.^17^

For cognitive interviews, 5-6 respondents are required for small-scale research. While various methods can be employed to conduct cognitive interviews, for this particular study concurrent verbal probing technique was employed to eliminate recall bias.^18^ For initial scale development, 25-40 participants are considered to be a reasonable sample for the pilot study.19 For this particular study, 30 participants were sent paper copies of the questionnaire out of which 27 returned the completed questionnaires. The Cronbach’s alpha for the tool was 0.87 which is within the acceptable range.20

The Integrated Curriculum Implementation Challenges (ICIC) is a useful instrument for identifying the challenges faced by faculty while implementing IC. It is an important and relevant instrument as most of the colleges in Pakistan are shifting from a traditional to IC due to the guidelines issued by PM&DC and no similar instrument exists in the literature. Hence, if the faculty’s perspective is considered, challenges identified and corrective measures taken timely, the implementation phase can be improved to make it smooth and less challenging.

### Limitations

It was a single-center study. Once more dental colleges implement IC, a cross-institutional study can be planned for a better comparison of faculty perspectives in terms of implementation challenges.

## CONCLUSION

ICIC is a useful instrument with good reliability and content validity. It can be used to identify the presence and extent of challenges faced by the faculty while implementing an integrated curriculum.

### Authors Contribution:

**KA:** Conception & design, data collection & analysis, manuscript writing, responsible for the integrity of research

**RAK:** Design, reviewed manuscript for approval.

**MA, FZZ:** Data collection, manuscript writing & editing.

## References

[1] Schneiderhan J, Guetterman TC, Dobson ML (2019). Curriculum development:A how to primer. Fam Med Community Health.

[2] Whyte B, Farser D, Aitken V (2013). Connecting Curriculum, Linking Learning. New Zealand Council Educ Res.

[3] Nagdeo N (2014). Integrated teaching. J Educ Technol Health Sci.

[4] Iqbal K, Asif S, Rehman IU (2017). BDS Curriculum:Neglected Sibling. J Pak Dent Assoc.

[5] Dreyer WP (2000). Dental education at the crossroads. S Afr Dent J.

[6] Reis S (2018). Curriculum reform:Why?What?How?and how will we know it works?. Isr J Health Policy Res.

[7] Sheikh QM, Chaudhry MWG, Masood F, Masood M, Mustafa G, Ijaz A (2020). Dental Curriculum Development By Engaging Students:the Need of Time. Pak Armed Forces Med J.

[8] Akram A, Rizwan F, Sattar K, Hadi JIS, Meo SA (2018). An approach for developing integrated undergraduate medical curriculum. Pak J Med Sci.

[9] Artino AR, La Rochelle JS, Dezee KJ, Gehlbach H (2014). Developing questionnaires for educational research:AMEE Guide No. 87. Med Teach.

[10] Cooper T (2017). Curriculum Renewal:Barriers to Successful Curriculum Change and Suggestions for Improvement. J Educ Train Stud.

[11] Gopalakrishnan S, Catherine AP, Kandasamy S, Ganesan H (2022). Challenges and opportunities in the implementation of competency-based medical education - A cross-sectional survey among medical faculty in India. J Educ Health Promot.

[12] Cooper, E (2009). Overloading on Slides:Cognitive Load Theory and Microsoft's Slide Program PowerPoint. AACE Review (formerly AACE J).

[13] Ali S.K, Baig L.A (2012). Problems and issues in implementing innovative curriculum in the developing countries:the Pakistani experience. BMC Med Educ.

[14] Schwartz A.R, Siegel M.D, Lee A.I (2019). A novel approach to the program evaluation committee. BMC Med Educ.

[15] Pessoa TRRF, Noro LRA (2020). Training in Dentistry:challenges for teacher development and effective inclusion in the Unified Health System. Revista Da ABENO.

[16] Sandhu D (2019). Healthcare educational leadership in the twenty-first century. Med Teach.

[17] Polit DF, Beck CT (2006). The content validity index:are you sure you know what's being reported?Critique and recommendations. Res Nurs Health.

[18] Karabenick SA, Woolley ME, Friedel JM, Ammon B V, Blazevski J, Bonney CR (2007). Cognitive Processing of Self-Report Items in Educational Research:Do They Think What We Mean?. Educ Psychol.

[19] Johanson GA, Brooks GP (2009). Initial Scale Development:Sample Size for Pilot Studies. Educ Psychol Meas.

[20] Tavakol M, Dennick R (2011). Making sense of Cronbach's alpha. Int J Med Educ.

